# Cystatin from the helminth *Ascaris lumbricoides* upregulates mevalonate and cholesterol biosynthesis pathways and immunomodulatory genes in human monocyte-derived dendritic cells

**DOI:** 10.3389/fimmu.2024.1328401

**Published:** 2024-02-28

**Authors:** Nathalie Acevedo, Ana Lozano, Josefina Zakzuk, Kevin Llinás-Caballero, David Brodin, Peter Nejsum, Andrew R. Williams, Luis Caraballo

**Affiliations:** ^1^ Institute for Immunological Research, University of Cartagena, Cartagena, Colombia; ^2^ Bioinformatics and Expression Analysis Core Facility (BEA), Karolinska Institutet, Huddinge, Sweden; ^3^ Department of Clinical Medicine. Aarhus University, Aarhus, Denmark; ^4^ Department of Veterinary and Animal Sciences. University of Copenhagen, Frederiksberg, Denmark

**Keywords:** cystatin, dendritic cells, immunomodulation, mevalonate pathway, transcriptome

## Abstract

**Background:**

*Ascaris lumbricoides* cystatin (Al-CPI) prevents the development of allergic airway inflammation and dextran-induced colitis in mice models. It has been suggested that helminth-derived cystatins inhibit cathepsins in dendritic cells (DC), but their immunomodulatory mechanisms are unclear. We aimed to analyze the transcriptional profile of human monocyte-derived DC (moDC) upon stimulation with Al-CPI to elucidate target genes and pathways of parasite immunomodulation.

**Methods:**

moDC were generated from peripheral blood monocytes from six healthy human donors of Denmark, stimulated with 1 µM of Al-CPI, and cultured for 5 hours at 37°C. RNA was sequenced using TrueSeq RNA libraries and the NextSeq 550 v2.5 (75 cycles) sequencing kit (Illumina, Inc). After QC, reads were aligned to the human GRCh38 genome using Spliced Transcripts Alignment to a Reference (STAR) software. Differential expression was calculated by DESEq2 and expressed in fold changes (FC). Cell surface markers and cytokine production by moDC were evaluated by flow cytometry.

**Results:**

Compared to unstimulated cells, Al-CPI stimulated moDC showed differential expression of 444 transcripts (|FC| ≥1.3). The top significant differences were in Kruppel-like factor 10 (*KLF10*, FC 3.3, P_BH_ = 3 x 10-^136^), palladin (FC 2, P_BH_ = 3 x 10-^41^), and the low-density lipoprotein receptor (*LDLR*, FC 2.6, P_BH_ = 5 x 10-^41^). Upregulated genes were enriched in regulation of cholesterol biosynthesis by sterol regulatory element-binding proteins (SREBP) signaling pathways and immune pathways. Several genes in the cholesterol biosynthetic pathway showed significantly increased expression upon Al-CPI stimulation, even in the presence of lipopolysaccharide (LPS). Regarding the pathway of negative regulation of immune response, we found a significant decrease in the cell surface expression of CD86, HLA-DR, and PD-L1 upon stimulation with 1 µM Al-CPI.

**Conclusion:**

Al-CPI modifies the transcriptome of moDC, increasing several transcripts encoding enzymes involved in cholesterol biosynthesis and SREBP signaling. Moreover, Al-CPI target several transcripts in the TNF-alpha signaling pathway influencing cytokine release by moDC. In addition, mRNA levels of genes encoding *KLF10* and other members of the TGF beta and the IL-10 families were also modified by Al-CPI stimulation. The regulation of the mevalonate pathway and cholesterol biosynthesis suggests new mechanisms involved in DC responses to helminth immunomodulatory molecules.

## 1 Introduction

Soil-transmitted helminth (STH) infections are among the most common infections worldwide with an estimated 1.5 billion infected people, with the highest prevalence reported from sub-Saharan Africa, China, South America, and Asia (1). The nematode *Ascaris lumbricoides* is the main STH infecting humans; it is a roundworm with a complex lifecycle, that after hatching from its egg as a microscopic larva enters the intestine and migrates to the liver and then to the lung before finally completing its cycle as an adult worm in the intestine (2). Ascaris infection has a dual effect on the immune system: it promotes allergic sensitization and induces immunomodulation (3–7). As a parasite, *Ascaris* has developed several evasion mechanisms supported by molecular and cellular mechanisms including the secretion of immunomodulatory molecules including cystatins (8, 9).

Cystatins are cysteine protease inhibitors that bind to cysteine proteases and regulate their proteolytic activities (8). Previously, we have observed that *Ascaris lumbricoides* cystatin (Al-CPI) prevents the development of dextran-induced colitis (10) and allergic airway inflammation in mouse models (11). Cystatins from helminth parasites can modulate host immune systems through different mechanisms (12). For instance, filarial cystatin (Bm-CPI-2) is known to inhibit the presentation of tetanus toxin by inhibiting the cathepsins necessary for antigen presentation via MHC-II (13). We also found that Al-CPI reduces the expression of co-stimulatory molecules CD86 and CD83 in LPS-activated monocyte-derived dendritic cells (moDCs) (11). Moreover, bone marrow dendritic cells generated in the presence of a cystatin from *Heligmosomoides polygyrus* (rHp-CPI) exhibited reduced expression of CD40, CD86, and MHC-II molecules (14).

Together with other immune cells (15–21), dendritic cells (DCs) are critical cell targets of parasite cystatins, and several studies have confirmed that they inhibit cathepsins L and S, DC differentiation, cytokine secretion, and alter inflammatory DC signaling (22–25). Indeed, the internalization of parasite cystatins is considered an evasive mechanism that reduces the expression of MHC class II molecules and CD86 (25). These immunomodulatory properties of parasite cystatins could facilitate parasite survival because in the infection model with the intestinal roundworm *Nippostrongylus brasiliensis*, mice with anti-nippocystatin antibodies were partially resistant to the infection (26).

The internalization of cystatins by immune cells especially monocytes, macrophages, and DCs is particularly relevant due to its implication on the antigen presenting machinery (27–29). Mammalian hosts also have cystatins that regulate endogenous cathepsins in DCs (30–33) and overexpression of cystatin C in bone marrow DCs reduces their capacity to stimulate CD4^+^ T-cell proliferation and decreases major histocompatibility complex-II presentation through a decrease in chaperon H2-DM (34). Cystatin C can also induce polarization of T cell differentiation to a regulatory phenotype (35). Although most effects of cystatins have been attributed to their enzymatic activities (9, 13, 21, 26), their mechanisms of immunomodulation are not well understood, and beyond their known enzymatic activities, it is possible that cystatins can also affect signal transduction, cell metabolism, or even be recognized by ligands or cell surface sensors. Experimental models also suggest that cystatin effects as an immunomodulatory molecule may vary depending on whether it is administered during basal non-inflammatory conditions or in the presence of inflammation (e.g., when co-administered with LPS) (11).

Previous studies have shown that the Ascaris body fluid (ABF) induces genomewide and significant changes in gene expression in moDC, suggesting new genes involved in the immune readouts observed in functional assays (36, 37). Transcriptome analysis is a *hypothesis-free* tool for the unbiased discovery of pathways and molecules that mediate the biological effects of immunomodulators (38). At present, the specific transcriptional changes induced by purified cystatin remain unknown. Considering that cystatin is found in the excretory/secretory products of the ABF but at lower concentrations compared to other abundant antigens such as ABA-1 or the heme-binding protein (39, 40), we produced a recombinant cystatin from *A. lumbricoides* with enzymatic activity and undetectable endotoxin for stimulation analysis. The aim of this study was to analyze the transcriptional profile of human moDC upon stimulation with Al-CPI to elucidate genes and pathways that are targets of parasite immunomodulation.

This study discovered that Al-CPI modifies mRNA expression levels of several genes encoding enzymes involved in the synthesis of lipids such as mevalonate, isopentenyl-5-pyrophosphate, dimethylallyl-PP, squalene, zymostenol, 7-dehydrocholesterol, and finally cholesterol. Previous studies have found that mevalonate and isoprenoids are critical for DC function and cytokine production (41–43). We hypothesize that these changes induced by Al-CPI in the mevalonate pathway may affect DC function in favor of the parasite. Indeed, there is evidence that cholesterol elicits immunosuppression by activating myeloid-derived suppressor cells (44) and that intracellular cholesterol trafficking in DCs is important for optimal antigen presentation (45). In this study, we found that Al-CPI stimulation induces the upregulation of gene expression in the transcripts for 3-hydroxy-3-methylglutaryl-CoA synthase 1 (*HMGCS1*), 3-hydroxy-3-methylglutaryl-CoA reductase (*HMGCR*), mevalonate kinase (*MVK*), mevalonate diphosphate decarboxylase (*MVD*), isopentenyl-diphosphate delta isomerase 1 (*IDI1*), sterol-C5-desaturase (*SC5D*) encoding enzymes involved in lipid metabolism, and in the gene for low-density lipoprotein receptor (*LDLR*). Our findings suggest that *A. lumbricoides* cystatin regulates DC function through changes in the expression of mevalonate and cholesterol-related genes.

## 2 Materials and methods

### 2.1 *Ascaris lumbricoides* cystatin Al-CPI

**Figure 1:**
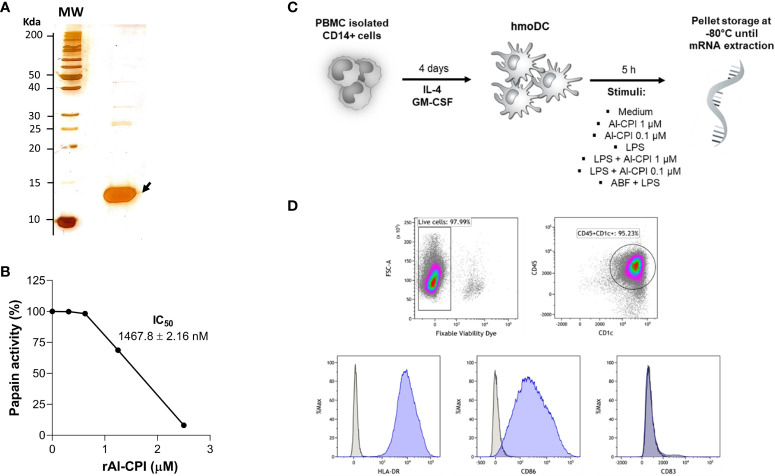
**(A)** Silver stain of purified Al-CPI cystatin used in this study. **(B)** Enzymatic activity of Al-CPI on papain. **(C)** Overview of the experimental conditions in which moDC were obtained from monocytes and stimulated by two concentrations of Al-CPI in the presence or absence of LPS or the Ascaris body fluid (ABF). **(D)** Immunophenotype of isolated moDC as CD45^+^ CD1c^+^ HLA-DR^+^ CD86^+^.

The sequence of *A. lumbricoides* cystatin (Al-CPI, GenBank ADR51550.1) without signal peptide nor histidine tag was cloned in pET15b (GenScript, USA) and expressed in *Escherichia coli* BL21 (DE3). The recombinant protein was obtained at >99% of purity by fast performance liquid chromatography (BiologicDuoFlowTM, Bio-Rad) using an anion exchange column (UNO Q1) and verified by silver stain in SDS-PAGE as a protein of 13 kDa (**Figure 1A**). Endotoxin was removed using ToxinEraserTM and verified by ToxinSensorTM Chromogenic LAL following the manufacturer’s instructions (GenScript, Piscataway, USA). LPS was obtained from Sigma-Aldrich (product code L2654) and the ABF from adult *A. suum* worms as previously described (46).

### 2.2 Biological activity of Al-CPI

The enzymatic activity of Al-CPI was analyzed using the protease inhibition assay of papain (Sigma-Aldrich, N° Cat. P41762) on its substrate p-Glu-Phe-Leu-p-nitroanilide diluted in water (**Figure 1B**) as previously described (10). In brief, kinetics of protease inhibition activity of Al-CPI were assessed on cathepsin B and papain using specific colorimetric substrates (Z-Arg-Arg-p-nitroanilide and p-Glu-Phe-Leu-p-nitroanilide). In a 15-minute reaction, proteases and their specific substrates were incubated alone, with chicken cystatin (as positive control), or with serially diluted Al-CPI. Papain (50 μg/reaction) was activated in the assay buffer (50 mM Tris-HCl pH 7.6, containing 2 mM cysteine and 0.1 mM EDTA, pH 6.5) for 30 minutes at 40°C, and the papain substrate was diluted in DMSO. Cathepsin B (1 ng/reaction) was activated in assay buffer (100 mM sodium acetate, 2 mM EDTA, 1 mM DTT, pH 5.5) containing 4 mM cysteine for 5 minutes at room temperature, being the substrate diluted in water. Absorbance of the released products was detected using a spectrophotometer (SpectraMax) at 410 nm every 20 seconds for 5 minutes. The inhibitory activity of Al-CPI was expressed as a percentage of the total activity detected without the recombinant.

### 2.3 Preparation of monocyte-derived dendritic cells

Peripheral blood mononuclear cells were isolated from buffy coats from six healthy human donors from the Copenhagen area in Denmark by using Ficoll separation. Monocytes were isolated by positive selection using anti CD14 antibodies by magnetic associated cell sorting (MACS, Miltenyi). After viability evaluation by exclusion of trypan blue dye, monocytes were seeded at 1.25 x 10^6^ cells/mL in R10 medium (RPMI medium with 10% FBS and 1% penicillin) and cultivated in a cell culture flask at 37°C in a humidified atmosphere containing 5% CO_2_ for 4 days in the presence of IL-4 and GM-CSF (both at a concentration of 12.5 ng/mL, R&D Systems) as previously described (47). An overview of cell stimuli and phenotype is given in **Figures 1C, D**.

### 2.4 Stimulation assays

moDC were resuspended at 2x10^6^/ml in RPMI medium, seeded in 24 well plates (10^6^ cells per well), and stimulated for 20 minutes with Al-CPI at concentrations of 0.1 µM or 1 µM or with 50 µg/mL ABF. For those wells to be stimulated with LPS (alone or Al-CPI + LPS), this was added at a final concentration of 10 ng/mL. Stimulated moDC were cultured for 5 hours at 37°C and the cell pellets were stored at -80°C.

### 2.5 RNA isolation

RNA was isolated with the RNAeasy kit (Qiagen, Hilden, Germany) following the manufacturer’s instructions. RLT buffer was added to the cells, loaded onto the RNAeasy spin minicolumn and the RNA eluted in RNA-free water. RNA quality was verified by gel electrophoresis. RNA concentration and purity were evaluated by Nanodrop (Thermo Fisher) with a mean concentration RNA level of 71.7 ng/µL and a mean A260/A280 ratio of 2.0.

### 2.6 RNA sequencing

Samples were normalized to 50 ng/µl and the RNAseq libraries were prepared using the mRNA seq Illumina TrueSeq and sequenced in a NextSeq 550 75 cycles v2.5. Sample quality was assessed using FastQC (v0.11.8) and MultiQC (v1.7). Reads were aligned to a reference built from Ensembl GRCh38 genome sequences using STAR (v2.6.1d). Counts for each gene were obtained using featureCounts (v1.5.1) using the GRCm38.101 GTF file from Ensembl. The bioconductor package DESEq2 was used for count normalization and sample group comparisons, generating log2 fold changes, Wald test p-values, and p-value adjustments for multiple testing (Benjamini-Hochberg method). To control for interindividual variability, the moDC donor was included as a covariate in the model to calculate differential expression.

### 2.7 Functional annotation and statistical analysis

Genes showing differential expression between Al-CPI stimulated and non-stimulated cells were analyzed by gene set enrichment analysis (GSEA) using the fgsea R package (v1.4) with MsigDB (v.7.4) (48) and network analysis using ConsensusPath DB (49). Principal component analysis (PCA) was performed using the prcomp function in R stats package (v4.2.1). Normalized transcript counts were compared among experimental groups using the Kruskal-Wallis test followed by the Conover-Iman test with Holm’s correction for multiple comparisons in DescTools (v0.99.49) (50). Enrichment, PCA, volcano, and violin plots were visualized using ggplot2 (v3.4.2) (51). Pathways were downloaded from the WikiPathways pathway collections and edited in PathVisio 3.3.0 (52).

### 2.8 Functional cell assays

moDCs were stained with the fixable viability dye eFluor 780 (eBiosciences, San Diego, CA, USA) for 20 min, followed by staining with anti-HLA-DR APC (clone G46-6, BD Biosciences), anti-CD83 FITC (clone HB15e, BD Biosciences), anti-CD86 PE-CF594 (clone 2331, BD Biosciences), anti-CD1c PerCP-eFluor™ 710 (clone L161, Thermo Fisher Scientific), and anti-CD45 BV510 (Clone HI30, BD Biosciences). Cytokines (IL-1β, IL-6, IL-8, IL-10, IL-12p70, and TNF) were measured in supernatants using the BD™ Human Inflammatory Cytokine Cytometric Bead Array (CBA), following the manufacturer’s instructions. Flow cytometry data were acquired using the FACS Aria III (BD, Franklin Lakes, USA) and analyzed with Kaluza, version 2.1 (Beckman Coulter Life Sciences, Indianapolis, USA).

## 3 Results

### 3.1 Gene expression differences between Al-CPI stimulated and non-stimulated moDC

**Figure 2:**
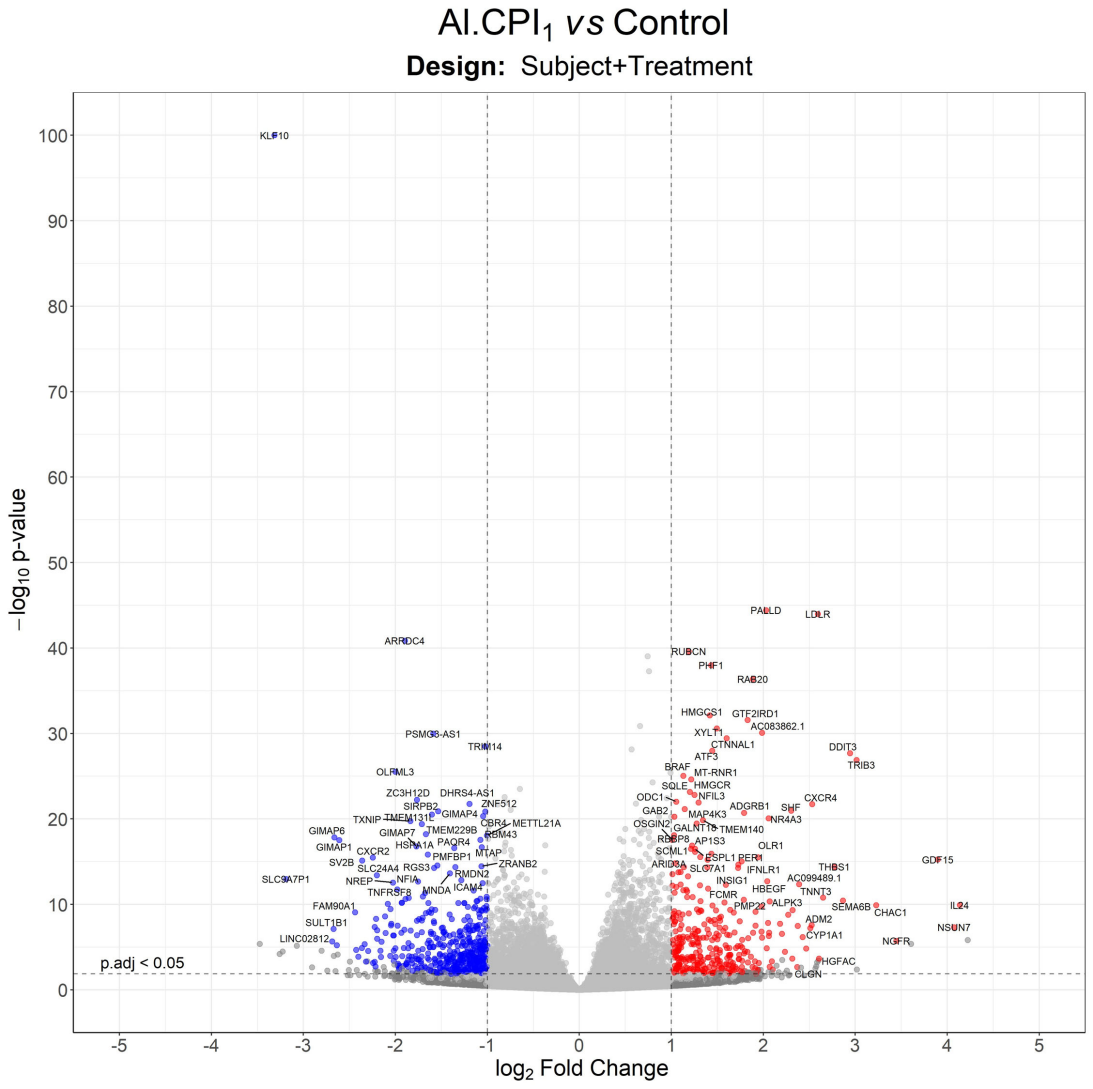
Volcano plot with differentially expressed genes between moDC stimulated with 1 µM Al-CPI compared to non-stimulated controls. Each dot represents a gene. Blue dots indicate genes downregulated by Al-CPI and red dots indicate upregulated genes.

**Figure 3:**
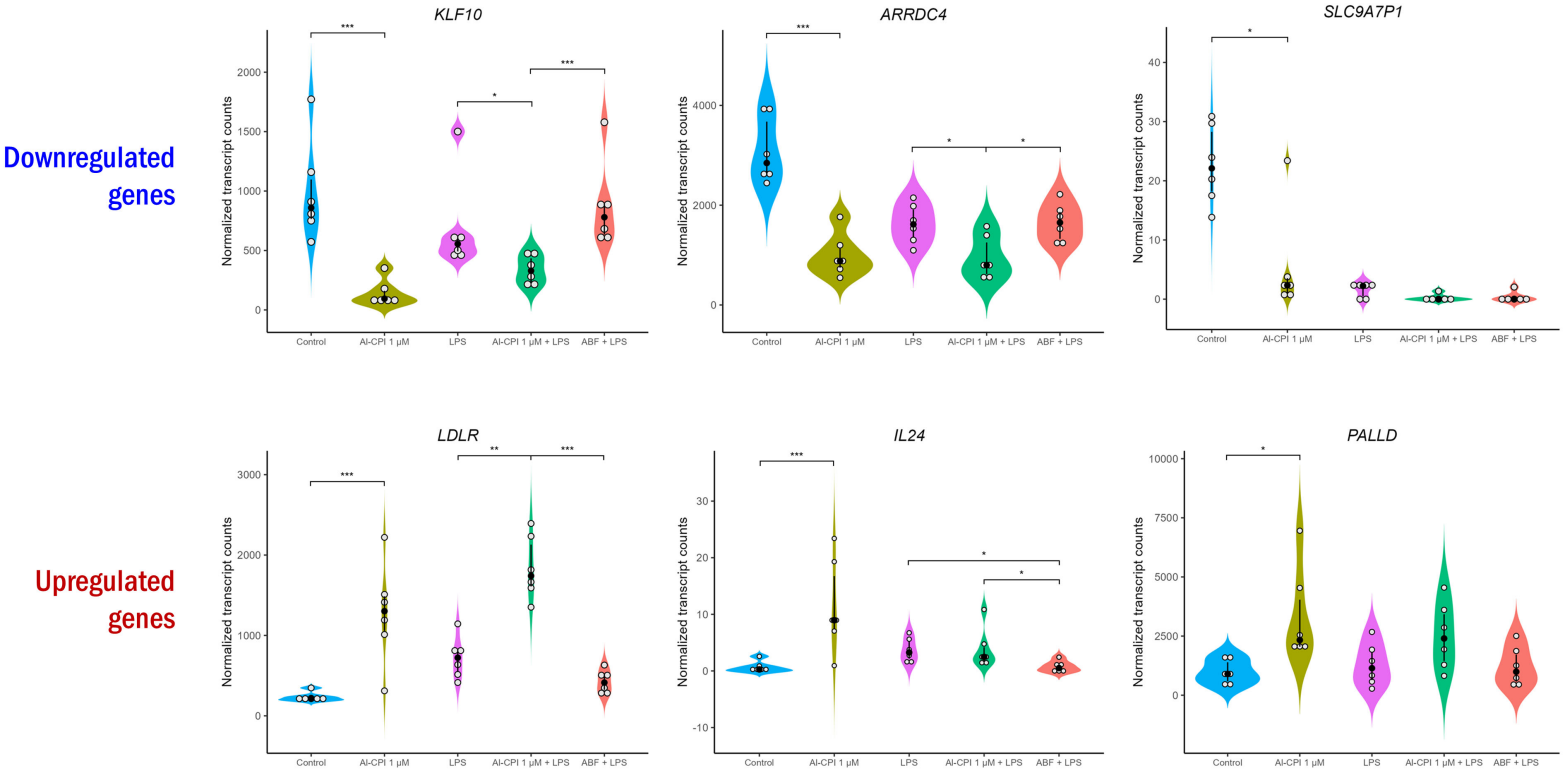
Normalized transcript levels of top significant differentially expressed genes upon stimulation with 1 µM Al-CPI. Transcript levels in the presence of LPS and ABF are presented as inflammatory and anti-inflammatory models, respectively. White circles represent individuals; black circles and lines represent the median and interquartile range, respectively. Significance levels are shown only for Control vs. 1 µM Al-CPI and LPS vs. 1 µM Al-CPI + LPS vs. ABF + LPS. KLF10, Kruppel-like factor 10; ARRDC4, arrestin domain containing 4; SLC9A7P1, solute carrier family 9-member 7 pseudogene 1; LDLR, low density lipoprotein receptor; IL24, interleukin 24; PALLD, palladin, cytoskeletal associated protein. *P < 0.05, **P < 0.01, ***P < 0.001.

To improve recombinant protein production and purification, Al-CPI was produced without his-tag and obtained with a high level of purity as verified by silver staining (**Figure 1A**). The expected inhibition of papain protease activity was also confirmed using a colorimetric method (**Figure 1B**). The biologically functional Al-CPI was added to moDC (**Figure 1C**) and log2 expression values were obtained from each donor (**Supplementary Figure S1**). When stimulated at 0.1 µM, the Al-CPI did not induce expression changes compared to non-stimulated controls except for a transcript encoding the Kruppel-like factor 10 (*KLF10*) which showed a decreased expression in Al-CPI stimulated cells when compared to non-stimulated controls (FC -1.27, adjusted P = 2.2 x10^-20^). However, when moDC were stimulated with Al-CPI at a concentration of 1 µM, there were significant differences in gene expression in 444 transcripts, of which 422 were protein coding transcripts and 16 were long non-coding RNAs (lncRNA). The top significant differences were in the genes encoding Kruppel-like factor 10 (*KLF10*, FC -3.3, PBH = 3 x 10-^136^) and the low-density lipoprotein receptor (LDLR, FC 2.6, P_BH_ = 5 x 10^-41^) (**Figure 2**). A summary of the top differentially expressed genes upon stimulation of 1 µM Al-CPI compared to non-stimulated controls is presented in **Figure 3**. Some of these genes also exhibited differential expression when moDCs were treated with co-administration of LPS + Al-CPI. Interestingly, for some genes, these effects differed between the purified Al-CPI and the ABF (**Figure 3**).

### 3.2 Differentially expressed genes upon Al-CPI stimulation without LPS are enriched in cholesterol metabolism and TNF signaling

**Figure 4:**
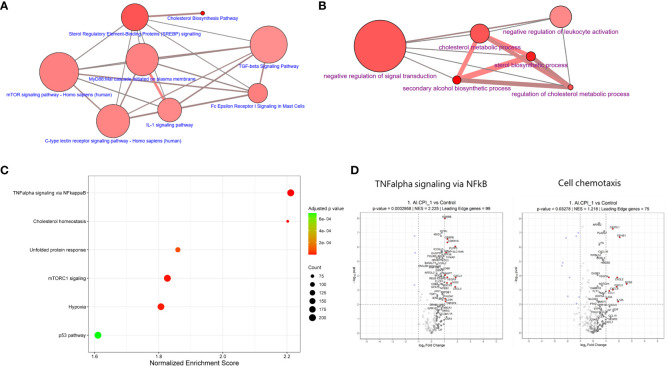
Functional annotation of enriched pathways upon Al-CPI stimulation. **(A)** Top biological pathways and **(B)** Gene ontology categories in which differentially expressed genes upon Al-CPI stimulation are overrepresented (false discovery rate < 0.01). **(C)** Most significantly enriched hallmark pathways (Human Molecular Signatures Database, MSigDB) resulting from GSEA adjusted by multiple testing. NES: normalized enrichment score. **(D)** Pathways enriched among differentially expressed genes involve molecules related with detectable differences in cytokine production. The leading-edge genes in the TNF-α signaling via NF-κB and cell chemotaxis are shown with non-transparent dots and also with labels if not too crowded. Significantly regulated genes are indicated in blue/red.

**Figure 5:**
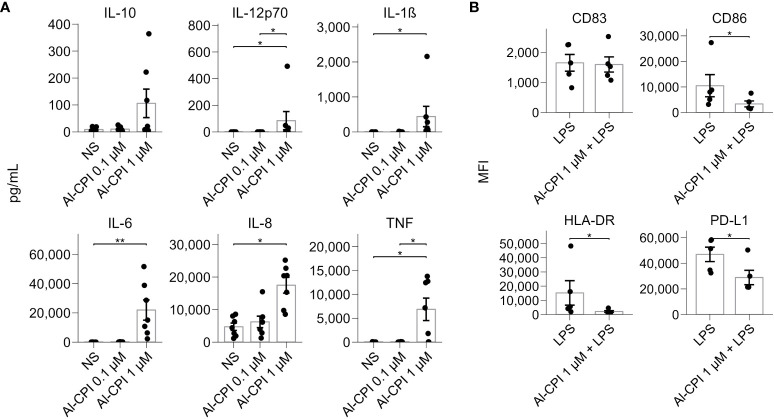
Cytokine production and cell surface markers upon ALCPI stimulation in moDCs. **(A)** Cytokine levels in moDC in culture supernatants upon stimulation of 0.1 μM or 1 μM of Al-CPI. *p<0.05, **p<0.01. NS: not stimulated. **(B)** Cell surface marker expression in the LPS inflammatory model with coadministration of Al-CPI. *p<0.05.

Functional annotation of differentially expressed genes revealed significant enrichment in sterol regulatory element-binding proteins (SREBP) signaling and cholesterol biosynthesis (**Figure 4A**). Gene ontology analysis also reported significant enrichment in cholesterol metabolic process and sterol biosynthesis (**Figure 4B**). The genes encoding lanosterol 14 alpha-demethylase (*CYP51A1)*, cholesterol 7 alpha-hydroxylase (*CYP7A1*), and 7-dehydrocholesterol reductase (*DHCR7*) were the most significant in the sterol biosynthetic process. GSEA revealed that the enrichment of differentially expressed genes in cholesterol metabolism was still significant after correction (P= 3.3 x 10^-7^) (**Figure 4C**). In addition, GSEA highlighted the significant enrichment of several genes involved in TNF-alpha signaling via NF-kB and cell chemotaxis (**Figure 4D**). These changes seem to occur simultaneously with the differential gene expression of loci involved in the negative regulation of leukocyte activation and negative regulation of signal transduction (see connecting edges in **Figure 4B**). In agreement with the observations at the transcriptional level, the stimulation of Al-CPI at a dose of 1 µM induced changes in cytokine production that were not observed with the 0.1µM dose (**Figure 5A**).

### 3.3 mRNA expression of genes enriched in cholesterol metabolic pathways are still significant even in the presence of inflammation

In agreement with the significant differences in the transcript expression of genes involved in the GO term “negative regulation of leukocyte activation” (**Figure 4B**), cell assays showed that moDC stimulation with 1µM Al-CPI in the presence of LPS resulted in significant decreases in the cell surface expression of CD86, HLA-DR, and PD-L1 (**Figure 5B**), although not in the levels of proinflammatory cytokines (**Supplementary Figure S2**).

**Figure 6:**
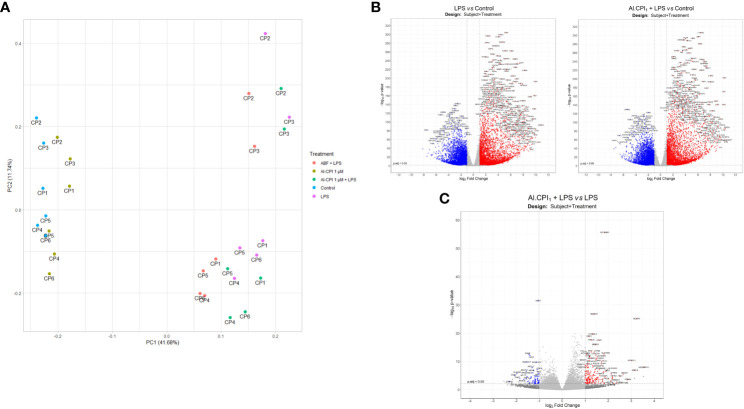
Gene expression differences in the LPS model with and without 1 μM Al-CPI stimulation. **(A)** Principal component analysis of normalized transcript counts between treatment groups. Each dot represents the moDC of a given patient (CP1-6) under different conditions. **(B)** Volcano plots with differentially expressed genes between moDC stimulated with LPS or 1 μM Al-CPI compared to non-stimulated controls. **(C)** Volcano plot with differentially expressed genes between moDC stimulated with LPS + 1 μM Al-CPI compared to LPS alone. Each dot represents a gene. Blue dots indicate downregulated genes and red dots indicate upregulated genes **(B, C)**.

The PCA plot shows a clear separation between samples treated and not treated with LPS in the first principal component (*x*-axis), the most important component in explaining data set variance, while the Al-CPI+LPS and LPS samples are closely entangled (**Figure 6A**). The effect of Al-CPI is also indicated in the volcano plots, where LPS heavily induces expression changes (**Figure 6B**) as compared to the effect of co-incubation with Al-CPI (**Figure 6C**). When LPS stimulated moDC were compared with LPS + 1 µM Al-CPI stimulated moDCk, it became evident that several genes encoding enzymes in the mevalonate and cholesterol synthesis pathways remained significant (**Figure 6C**). These include 3-hydroxy-3-methylglutaryl-CoA reductase (*HMGCR*), mevalonate kinase (*MVK*), fatty acid desaturase 1 (*FADS1*), and sterol-C5-desaturase (*SC5D*) involved in lipid metabolism, and others involved in the formation of actin filaments (palladin) and with actin binding activity (*CTNNAL1*). The upregulation of *LDLR* in the presence of Al-CPI is still observed in the inflammatory model with LPS (see volcano plot in **Figure 6C**).

**Figure 7:**
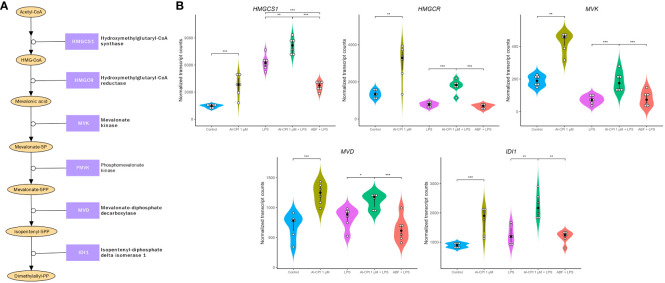
Gene expression differences in the mevalonate pathway in moDC upon stimulation with 1 μM Al-CPI. Effects on transcription are detectable either alone or in combination with LPS. **(A)** Diagram of the mevalonate pathway. Enzymes with significant expression differences between groups are represented in bold. **(B)** Violin plots comparing the expression levels of five genes in the mevalonate pathway. White circles represent individuals; black circles and lines represent the median and interquartile range, respectively. Significance levels are shown only for Control vs. 1 μM Al-CPI and LPS vs. 1 μM Al-CPI + LPS vs. ABF + LPS. HMGCS1, 3-hydroxy-3-methylglutaryl-CoA synthase 1; HMGCR, 3-hydroxy-3-methylglutaryl-CoA reductase; IDI1, isopentenyl-diphosphate delta isomerase 1; MVD, mevalonate diphosphate decarboxylase. *P < 0.05, **P < 0.01, ***P < 0.001.

**Figure 8:**
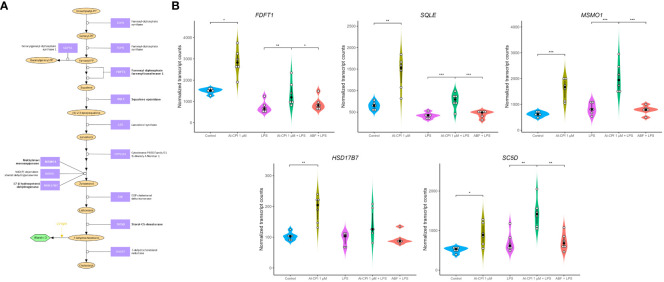
Differential gene expression in molecules involved in cholesterol biosynthesis in moDC upon stimulation with 1 μM Al-CPI. Effects on transcription are detectable either alone or in combination with LPS. **(A)** Diagram of cholesterol biosynthesis downstream of the mevalonate pathway. Enzymes with significant expression differences between groups are represented in bold. Note that cholesterol can also be synthesized from lanosterol through other pathways involving mostly the same enzymes shown here. **(B)** Violin plots comparing expression levels of five genes in the cholesterol biosynthesis pathway. White circles represent individuals; black circles and lines represent the median and interquartile range, respectively. Significance levels are shown only for Control vs. 1 μM Al-CPI and LPS vs. 1 μM Al-CPI + LPS vs. ABF + LPS. FDFT1, farnesyl-diphosphate farnesyltransferase 1; SQLE, squalene epoxidase; MSMO1, methylsterol monooxygenase 1; HSD17B7, hydroxysteroid 17-beta dehydrogenase 7; SC5D, sterol-C5-desaturase. *P < 0.05, **P < 0.01, ***P < 0.001.

In addition to the Al-CPI stimulation with and without LPS, we also performed comparative analysis with LPS and with ABF. mRNA expression of genes involved in the mevalonate pathway, including 3-hydroxy-3-methylglutaryl-CoA synthase 1 (*HMGCS1*), 3-hydroxy-3-methylglutaryl-CoA reductase (*HMGCR*), mevalonate kinase (*MVK*), mevalonate diphosphate decarboxylase (*MVD*), and isopentenyl-diphosphate delta isomerase 1 (*IDI1*), were upregulated upon Al-CPI stimulation alone or when comparing LPS + Al-CPI 1 μM versus LPS alone (**Figure 7**). Other genes involved in the downstream pathway of dimethylallyl pyrophosphate, cholesterol biosynthesis, and steroid metabolism also showed differential expression: farnesyl-diphosphate farnesyltransferase 1 (*FDFT1*), squalene epoxidase (*SQLE*), methylsterol monooxygenase 1 (*MSMO1*), hydroxysteroid 17-beta dehydrogenase (*HSD17B7*), and sterol-C5-desaturase (*SC5D*) (**Figure 8**). Notably, the effects of Al-CPI on transcription are maintained or in some cases even increased in the presence of LPS and were not achieved with the ABF. In addition, the expression of genes involved in anti-inflammatory pathways (e.g., CTLA4 and *ARG2*) and TNF signaling was modified in moDCs treated with Al-CPI (**Supplementary Figure S3**).

## 4 Discussion

**Figure 9:**
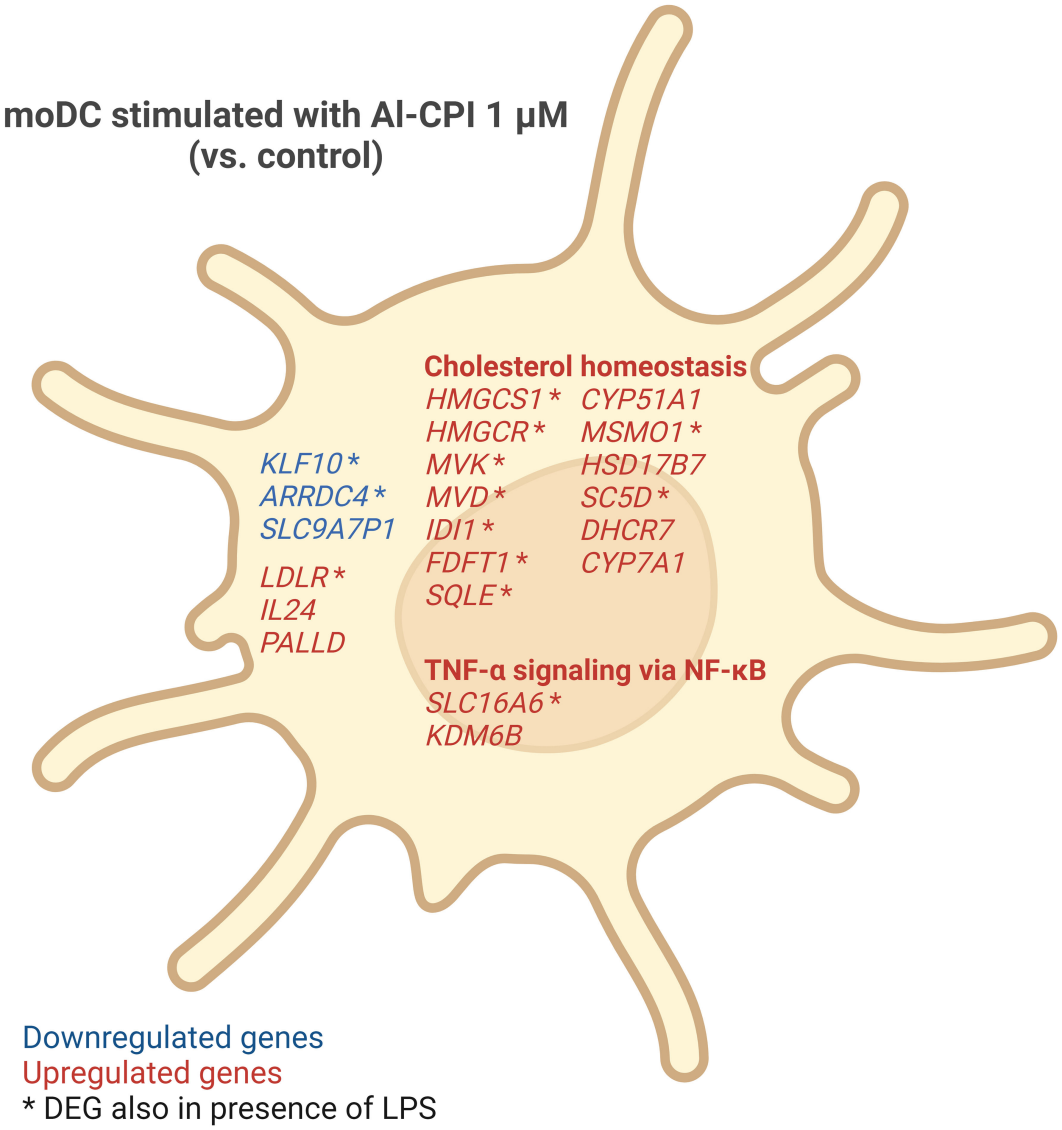
Summary of gene expression changes in moDC induced by 1 μM Al-CPI. Cholesterol homeostasis and TNF-α signaling via NF-κB pathways were significantly enriched among DEGs. Some genes (indicated by asterisks) showed differential expression even in the presence of LPS and Al-CPI simultaneously.

This study shows for the very first time the transcriptional changes that occur in moDC upon stimulation of a purified parasitic molecule (**Figure 9**). Remarkably, we found that Al-CPI induces the differential gene expression of several enzymes involved in the sterol biosynthetic process and genes activated by the SREBP. This pathway is a metabolic route that converts acetyl-CoA to 3-hydroxy-3-methyl-glutaryl-CoA and then mevalonic acid and mevalonate (**Figure 7**). Interestingly, mevalonate is a precursor of cholesterol as well as isoprenoid intermediates such as farnesyl pyrophosphate and geranyl pyrophosphate with critical roles in innate immunity and T cell function (53). The increased expression of genes in the cholesterol biosynthetic pathway was even observed when Al-CPI was incubated with LPS (**Figure 8**).

Previous studies have shown that DCs require the mevalonate pathway for effector cytokine production (54) and that the mevalonate pathway is induced during myelopoiesis (55). In this study, the expression of *MVK* and *MVD* encoding mevalonate kinase and mevalonate diphosphate decarboxylase were increased in moDC treated with Al-CPI (**Figure 7**); previous studies have shown that the resulting compound of this reaction, isopentenyl diphosphate (IPP), can be exported to the extracellular space by DCs using the cholesterol efflux transporter ABCA1 and this serves as an indicator of hyperactive mevalonate metabolism that alerts the immune system (56). The enrichment of differentially expressed genes in the mTOR pathway in connection with sterol regulatory elements binding proteins (**Figures 3A, C**) is also interesting and in line with recent reports showing that the mTOR-driven metabolic reprogramming of macrophages increased flux through the mevalonate pathway and this process is involved in trained immunity (57). In this study, the *LDLR* gene encoding for the LDL receptor was the top significant upregulated gene and more studies are needed to evaluate its role as a cell surface sensor or innate receptor in response to Al-CPI or whether it is implicated in the transcriptional changes of sterol metabolic genes.

Lipid metabolism is crucial in DCs maturation and activation; changes in this process can alter DCs function and in some instances induce tolerogenic cells (58). For example, accumulation of intracellular oxidized lipids (cholesterol, triglycerides, and fatty acids) induced by tumor-derived factors impacts antigen presentation in human moDCs and mouse DCs (59, 60). It has been demonstrated that cholesterol regulatory pathways in DCs regulate their maturation into immunogenic or tolerogenic DCs (61). In line with our findings regarding the overexpression of genes regulated by SREBP2, recent data show that this pathway is critical for the development and tolerogenic function of regulatory DCs in the tumor microenvironment (41).

Given that cholesterol is important for DC homeostasis and function, we speculate that the observed changes in the expression of genes related to cholesterol metabolism in DCs may serve as a mechanism to modulate the immune response in favor of parasite survival. For instance, Zika virus infection of moDC increases the expression of genes involved in cholesterol biosynthesis (62), several of which were also upregulated in this study upon exposure to 1 µM Al-CPI. The Zika virus modulates DC function by modifying the expression of the SREBP2-dependent cholesterol biosynthetic pathway, highlighting its importance in the adequate functioning of these cells (62). Moreover, membrane cholesterol is required for DC antigen presentation and their capacity to promote pathogen clearance (45, 63). Therefore, the helminth-derived immunomodulatory molecule Al-CPI may regulate DC function through their effects on lipid metabolism, including the differential expression of cholesterol-related genes.

In the functional bioinformatic annotation of differentially expressed genes upon Al-CPI stimulation, we found that “cholesterol metabolic process” and “regulation of cholesterol metabolic process” share genes with the “negative regulation of leukocyte activation” and “negative regulation of signal transduction” (shown as connecting edges in **Figure 4B**). We then measured cytokines and costimulatory molecules in the moDC as a readout of the latter pathways, finding that Al-CPI stimulation can increase cytokine production and decrease the expression of costimulatory molecules in the inflammatory model. However, a direct demonstration on the exact mechanism or metabolite that connects cholesterol metabolism with modulation of cytokines and costimulatory molecules in DCs needs to be formally demonstrated in future studies.

Intriguingly, the transcriptional changes in mevalonate and cholesterol genes remain in the LPS model. LPS is a potent stimulator of several inflammatory pathways (NF-kB, MAPK) and thereby a model to analyze the anti-inflammatory effect of Al-CPI to inhibit or modify gene expression. In this study, we observed that Al-CPI exerts a modest effect when co-administered in the *in vitro* LPS model (**Figure 6**); however, in both basal conditions (Al-CPI alone) or an inflammatory condition (LPS model), the Al-CPI stimulation resulted in consistent and significant differences in genes related to sterol metabolism (**Figures 7**, **8**). We also found that upon stimulation of 1µM Al-CPI in the presence of LPS, there were significant changes in the expression of immune cell markers such as CD86, HLA-DR, and PD-L1 involved in the pathways of negative regulation of immune response (**Figure 5B**). The fact that HLA-DR was significantly downregulated in the context of LPS in the cell stimulation assay but not at the transcript level suggests that other post-translational mechanisms may be implicated.

Other pathways related with immune response and immunomodulation were also significant (**Figures 4A–C**). These include TNF signaling via NF-kB and the TGF beta signaling already studied in the context of parasite immunomodulation and replicated in this transcriptomic screening (64). In relationship with the pathway of “negative regulation of leukocyte activation”, we here found that Al-CPI upregulated the expression of cytotoxic T-lymphocyte associated protein 4 (*CTLA4*) (**Supplementary Figure S3**), an inhibitory receptor acting as a major negative regulator of T-cell responses. This could play a role in possible mechanisms for immunomodulation explaining the inhibition of T cell proliferation upon stimulation with anti-CD2/CD3/CD28 when incubated with Al-CPI (65). We also detected increased expression in *ARG2* (**Supplementary Figure S3**), an enzyme involved in the downregulation of nitric oxide synthesis and immunomodulation (66–68). We here also found that Al-CPI effects depend on the donor of the DC, suggesting that some individuals are more amenable to cystatin immunomodulation.

The exact receptor of parasites cystatins and their effects on cell signaling are unclear. There is evidence that AvCystatin addresses the ERK- and p38-pathway to induce IL-10 and to regulate IL-12/23p40 production in macrophages (28). In our study, we found significant enrichment in the pathway “Myd88:Mal cascade initiated on plasma membrane” (**Figure 4A**) in agreement with recent reports suggesting that parasite cystatin can be sensed by toll-like receptor 4 (69) and that roundworm proteins can induce cytokine release upon TLR signaling (70). Other significant pathways identified in our study with differentially expressed genes upon Al-CPI stimulation include IL-1 signaling, C-type lectin receptor signaling, mTOR signaling, TGF beta signaling, and TNF signaling via NF-kB (**Figure 4**). More studies are needed to dissect the exact mechanism of how Al-CPI initiates and affects these pathways *in vivo*.

It can be speculated that transcriptional changes in the mevalonate and cholesterol genes may result when Al-CPI has been sensed by a cell surface receptor in the DC that activates the mTOR pathway. Our bioinformatic functional annotation revealed that Al-CPI differentially expressed genes are enriched in the mTOR signaling pathway (**Figures 4A, C**). Previous studies have demonstrated that mTORC1 activates sterol regulatory element-binding proteins (SREBPs) including SREBP-2, a master regulator of cholesterol synthesis. Through incompletely understood mechanisms, activated mTORC1 triggers translocation of SREBP-2, an endoplasmic reticulum (ER) resident protein, to the Golgi where SREBP-2 is cleaved to translocate to the nucleus and activate gene expression for cholesterol synthesis (71).

Our study had several strengths: first, it reports a transcriptome of viable moDC that were consistently differentiated and stimulated at two different doses of Al-CPI following well established protocols (47). The recombinant protein used here was FPLC purified without signal peptide or His-tag to resemble the native counterpart in human roundworm with verified enzymatic activity. The sequencing approach was very robust, and the statistical methods implemented applied the appropriate corrections to highlight the most consistent findings. We here also implemented two different doses for Al-CPI stimulation. To date, there are no studies showing the concentration of helminth cystatins that come in contact with DCs during *in vivo* infection, but the concentrations used in this study were titrated in the laboratory and found 1 μM Al-CPI as the optimal to induce cytokine response in moDC. Still, the gene *KLF10* which is a transcriptional repressor was significantly downregulated at both 0.1 μM and 1 μM, suggesting that some expression changes may occur at lower doses of Al-CPI. We also obtained transcriptomic data on moDC stimulated with only LPS and Al-CPI in the presence of LPS mimicking an inflammatory context. Still, this study has some limitations: first, we obtained moDC from six human donors and additional studies with a larger sample size will help to better control and analyze the effect of interindividual variability. Moreover, the transcriptome profile was obtained only at one time point after 5 hours of Al-CPI incubation (this was set to allow sufficient time between antigen stimulation and gene transcription, but before confounding with autocrine effects of antigen-induced cytokines); nevertheless, future studies are needed to elucidate the dynamics of gene expression at later time points upon Al-CPI stimulation. Finally, functional analysis to confirm the bioinformatic insights of this study are warranted.

In conclusion, Al-CPI modifies the transcriptome of moDC, increasing several transcripts encoding enzymes involved in cholesterol biosynthesis and SREBP signaling pathways. In addition, Al-CPI targets several transcripts involved in mTOR signaling and in the TNF signaling pathway, influencing cytokine release by moDC. mRNA levels of genes encoding *KLF10* and other members of the TGF beta and the IL-10 families are also modified by Al-CPI stimulation. The regulation of the mevalonate pathway and cholesterol biosynthesis suggests new mechanisms involved in DC responses to helminth immunomodulatory molecules.

## Data availability statement

The datasets presented in this study can be found in online repositories. The names of the repository/repositories and accession number(s) can be found below: GSE250463 (GEO).

## Ethics statement

Buffy coats were obtained from healthy human donors from the State Hospital (Copenhagen, Denmark), following informed, written consent in accordance with legislation and guidelines from the local ethics committee (Region Hovedstaden, Denmark). The studies were conducted in accordance with the local legislation and institutional requirements. The participants provided their written informed consent to participate in this study.

## Author contributions

NA: Conceptualization, Data curation, Formal Analysis, Funding acquisition, Investigation, Methodology, Visualization, Writing – original draft, Writing – review & editing. AL: Formal Analysis, Investigation, Methodology, Resources, Validation, Visualization, Writing – review & editing. JZ: Formal Analysis, Investigation, Methodology, Resources, Supervision, Validation, Visualization, Writing – review & editing. KL-C: Data curation, Methodology, Software, Visualization, Writing – review & editing. DB: Data curation, Formal Analysis, Investigation, Methodology, Software, Supervision, Visualization, Writing – review & editing. PN: Investigation, Resources, Supervision, Writing – review & editing. AW: Conceptualization, Investigation, Methodology, Project administration, Resources, Supervision, Writing – review & editing. LC: Conceptualization, Funding acquisition, Investigation, Methodology, Project administration, Resources, Supervision, Writing – original draft, Writing – review & editing.

## Supplementary material

Supplementary Figure S1 Distribution of the log2 expression levels of all detected transcripts across samples.

Supplementary Figure S2 Cytokine levels in the LPS inflammatory model with coadministration of Al-CPI. Error bars indicate standard error of the mean SEM. *p<0.05.

Supplementary Figure S3 Gene expression levels for cytotoxic T-lymphocyte associated protein 4 (*CTLA4*), arginase 2 (*ARG2*), solute carrier family 16 member 6 (*SLC16A6*), and lysine demethylase 6B (*KDM6B*) among treatment groups. White circles represent individuals; black circles and lines represent the median and interquartile range, respectively. Significance levels are shown only for Control vs. 1 μM Al-CPI and LPS vs. 1 μM Al-CPI + LPS vs. ABF + LPS. *p < 0.05, **p < 0.01, ***p<0.0001.
