# Supplementary figures and images for: Cystatin from the helminth Ascaris lumbricoides upregulates mevalonate and cholesterol biosynthesis pathways and immunomodulatory genes in human monocyte-derived dendritic cells
Source: Front Immunol. 2024 Feb 28;15:1328401. doi: 10.3389/fimmu.2024.1328401 (PMC10936004; doi:10.3389/fimmu.2024.1328401)

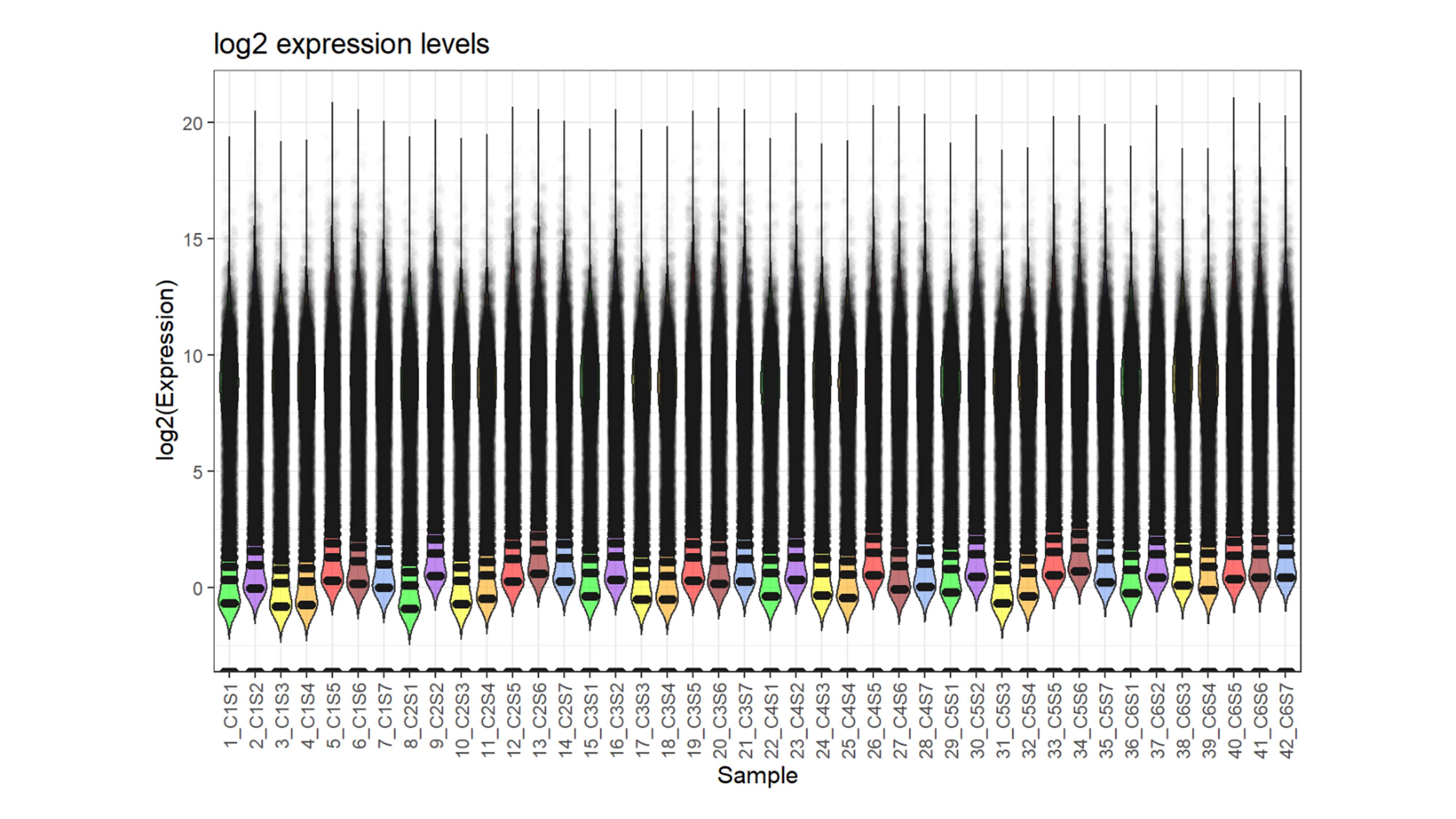

Supplement: Supplementary Figure S1 — Distribution of the log2 expression levels of all detected transcripts across samples. [file Image_1.tif]

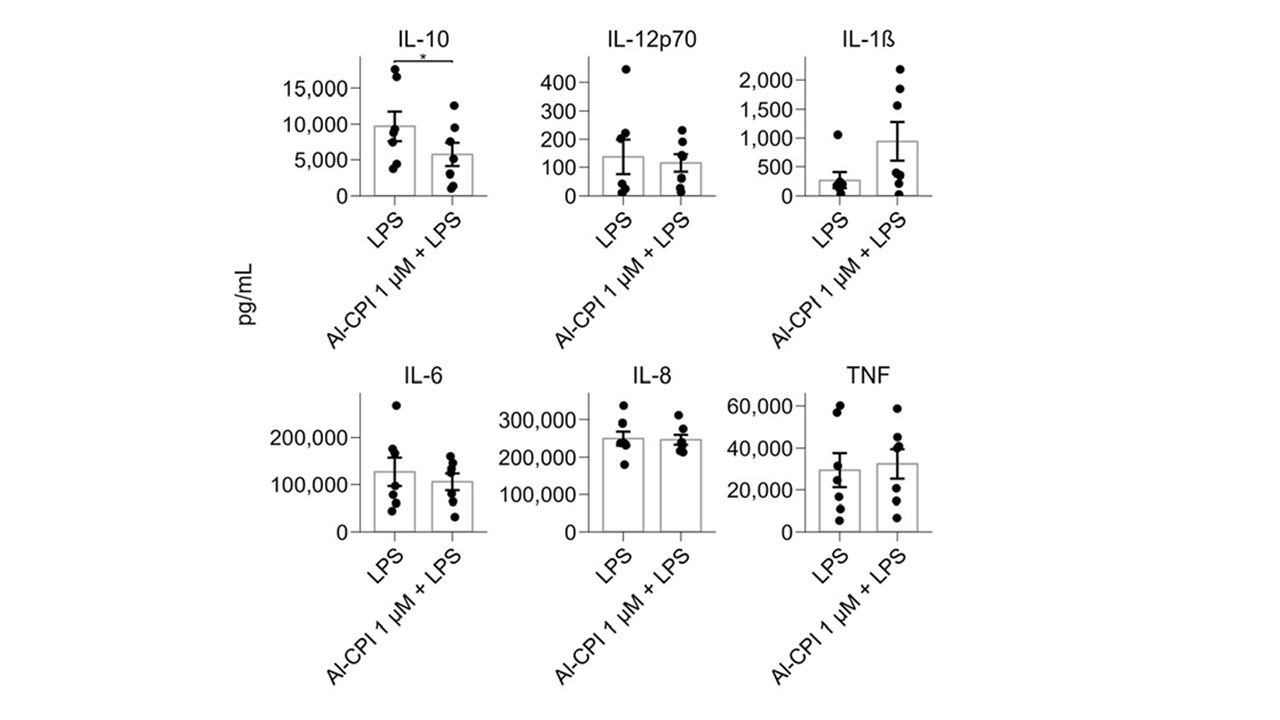

Supplement: Supplementary Figure S2 — Cytokine levels in the LPS inflammatory model with coadministration of Al-CPI. Error bars indicate standard error of the mean SEM. *p<0.05. [file Image_2.tif]

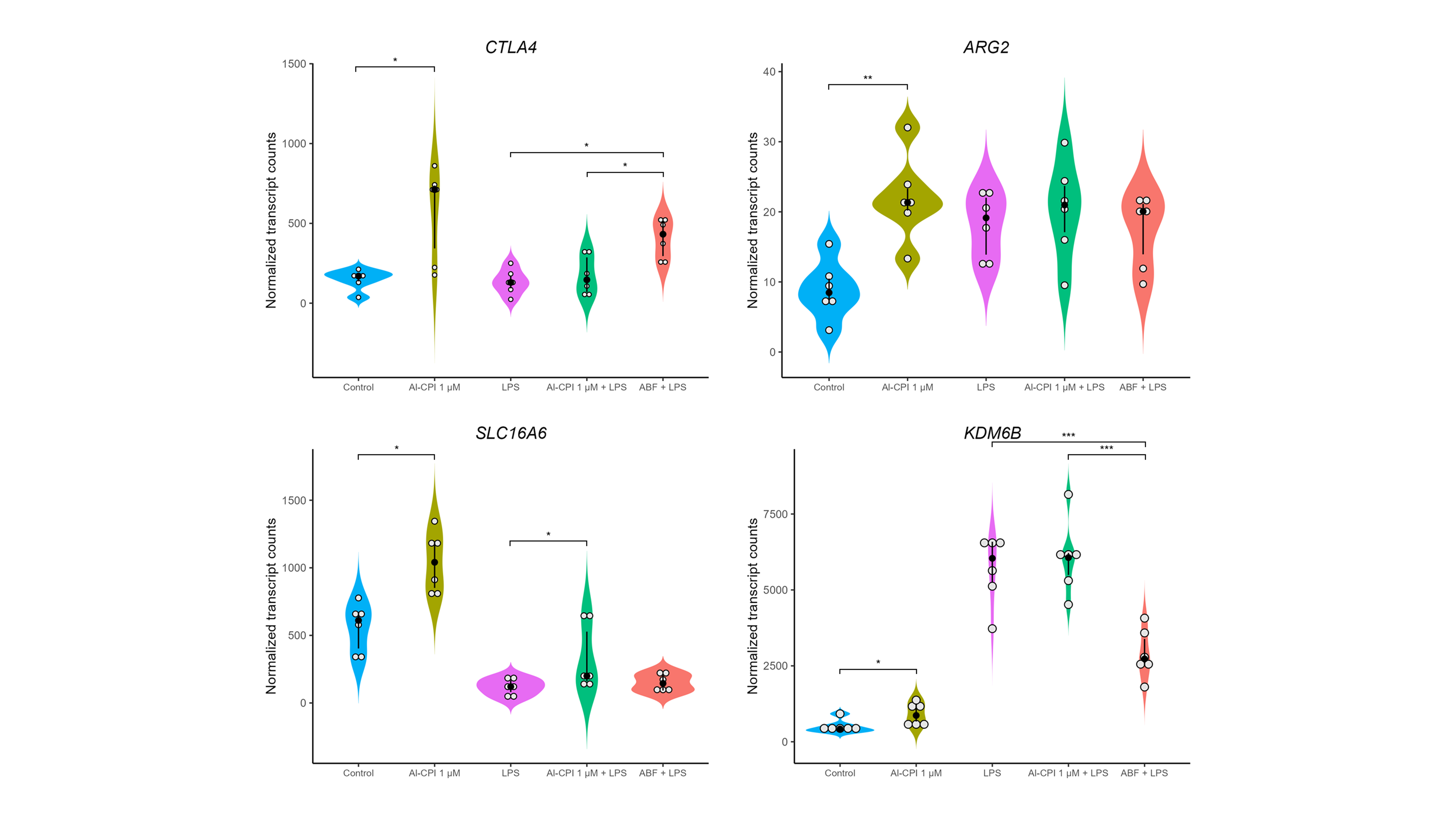

Supplement: Supplementary Figure S3 — Gene expression levels for cytotoxic T-lymphocyte associated protein 4 (CTLA4), arginase 2 (ARG2), solute carrier family 16 member 6 (SLC16A6), and lysine demethylase 6B (KDM6B) among treatment groups. White circles represent individuals; black circles and lines represent the median and interquartile range, respectively. Significance levels are shown only for Control vs. 1 μM Al-CPI and LPS vs. 1 μM Al-CPI + LPS vs. ABF + LPS. *p < 0.05, **p < 0.01, ***p<0.0001. [file Image_3.tif]
